# Lactose intolerance and gastrointestinal cow’s milk allergy in infants and children – common misconceptions revisited

**DOI:** 10.1186/s40413-017-0173-0

**Published:** 2017-12-12

**Authors:** Ralf G. Heine, Fawaz AlRefaee, Prashant Bachina, Julie C. De Leon, Lanlan Geng, Sitang Gong, José Armando Madrazo, Jarungchit Ngamphaiboon, Christina Ong, Jossie M. Rogacion

**Affiliations:** 1 Murdoch Childrens Research Institute, Melbourne, Australia; 2 Al Adan Hospital, Ministry of Health, Kuwait City, Kuwait; 3 Rainbow Children’s Hospital, Banjara Hills, Hyderabad, India; 4 Philippine Society of Allergy, Asthma & Immunology, Philippine Medical Association, Quezon City, Philippines; 5 Guangzhou Women and Children’s Medical Center, Guangzhou Medical University, Guangzhou, China; 6 Universidad National Autonoma de México, Hospital Infantil Privado Star Médica, Polanco, Mexico City, Mexico; 7 Chualalongkorn University, Bangkok, Thailand; 8 KK Women’s and Children’s Hospital and Yong Loo Lin School of Medicine and Duke-NUS Medical School, Singapore, Singapore; 9 University of the Philippines, Philippine General Hospital, Manila, Philippines

**Keywords:** Malabsorption, Carbohydrate, Enteropathy, Cow’s milk allergy, Celiac disease, Gastroenteritis

## Abstract

Lactose is the main carbohydrate in human and mammalian milk. Lactose requires enzymatic hydrolysis by lactase into D-glucose and D-galactose before it can be absorbed. Term infants express sufficient lactase to digest about one liter of breast milk daily. Physiological lactose malabsorption in infancy confers beneficial prebiotic effects, including the establishment of Bifidobacterium-rich fecal microbiota. In many populations, lactase levels decline after weaning (lactase non-persistence; LNP). LNP affects about 70% of the world’s population and is the physiological basis for primary lactose intolerance (LI). Persistence of lactase beyond infancy is linked to several single nucleotide polymorphisms in the lactase gene promoter region on chromosome 2. Primary LI generally does not manifest clinically before 5 years of age. LI in young children is typically caused by underlying gut conditions, such as viral gastroenteritis, giardiasis, cow’s milk enteropathy, celiac disease or Crohn’s disease. Therefore, LI in childhood is mostly transient and improves with resolution of the underlying pathology. There is ongoing confusion between LI and cow’s milk allergy (CMA) which still leads to misdiagnosis and inappropriate dietary management. In addition, perceived LI may cause unnecessary milk restriction and adverse nutritional outcomes. The treatment of LI involves the reduction, but not complete elimination, of lactose-containing foods. By contrast, breastfed infants with suspected CMA should undergo a trial of a strict cow’s milk protein-free maternal elimination diet. If the infant is not breastfed, an extensively hydrolyzed or amino acid-based formula and strict cow’s milk avoidance are the standard treatment for CMA. The majority of infants with CMA can tolerate lactose, except when an enteropathy with secondary lactase deficiency is present.

## Background

Lactose intolerance (LI) is a common gastrointestinal condition which is due to the inability to digest and absorb dietary lactose. Lactose requires hydrolysis by the enzyme lactase into D-glucose and D-galactose before it can be absorbed. About 70% of the world’s population suffer from LI due to a genetically programmed gradual decline in lactase expression after weaning, so-called lactase non-persistence (LNP) [1, 2]. The introduction of dairy farming and regular consumption of cow’s milk over 5000 years ago selected individuals who tolerated lactose-containing foods beyond early childhood [3]. Population genomics suggest that the ability to digest lactose beyond infancy (i.e. lactase persistence) emerged around the time of the Eurasian Bronze Age (3000–1000 B.C) [4]. While regular ingestion of milk and fermented milk products is likely to have improved individuals’ nutritional status, it remains unclear if other clinical benefits have promoted the genetic selection of lactase persistence [1].

LI presents with mild to moderate gastrointestinal symptoms, including abdominal pain, flatulence and diarrhea. Children under 5 years can generally tolerate lactose as primary LI rarely manifests clinically in this age group [5, 6]. However, in regions with a high prevalence of primary LI, the intake of cow’s milk-based products may be unnecessarily restricted. Due to the similarities in the clinical symptoms of gastrointestinal CMA and LI, there is ongoing diagnostic confusion - not only by parents but also amongst health professionals [7–9]. While transient lactose malabsorption following gastroenteritis is relatively common in children under 2 years [10, 11], more persistent symptoms due to cow’s milk enteropathy are often not recognized [12] and may be inappropriately treated with a lactose-free, cow’s milk protein-containing formula [7]. The following paper aims to provide an overview of the physiology, clinical presentation, differential diagnosis and treatment of LI. It will also address several common misconceptions about LI in infants and young children.

## Physiology of lactose absorption

Lactose (β-galactosyl-1,4 glucose) is the main carbohydrate in human and mammalian milk. Human milk contains about 7.5 g/100 mL of lactose, compared to about 5 g/100 mL in cow’s milk and other mammalian milk [13]. A term infant is typically able to digest about 60–70 g of lactose per day, equivalent to one liter of breast milk. Young infants do not absorb all of the ingested lactose from breast milk (physiological lactose malabsorption). Malabsorbed lactose is fermented in the colon to short-chain fatty acids (SCFA), hydrogen (H_2_), carbon dioxide (CO_2_) and methane (CH_4_). Malabsorbed lactose is also converted to lactic acid by enteric bacteria (*Streptococcus lactis* and others) [14].

### Lactase

Lactase-phlorizin hydrolase, commonly called lactase, splits lactose into D-glucose and D-galactose [15, 16]. Lactase is a member of the beta-galactosidase family and is only expressed by mature enterocytes, with its highest expression in the mid-jejunum [17]. The enzyme spans the apical membrane of mature enterocytes and is made up of two identical extracellular 160 kDa polypeptide chains, as well as a short intracytoplasmic part [18].

## Pathophysiology of lactose malabsorption

Individuals with LI absorb between 42 and 77% of ingested lactose after a 12.5 g dose, compared to 95% in lactase persisters [19]. The clinical manifestations of LI are due to osmotic fluid shifts into the gut, as well as gas formation and bowel distension. This may present with abdominal pain, flatulence and diarrhea. Several factors influence whether malabsorbed lactose will cause gastrointestinal symptoms, including the dose, food matrix, oro-cecal transit time and fermenting capacity of the fecal microbiota. In individuals with LI, ongoing lactose ingestion may ameliorate diarrhea and flatulence due to the proliferation of lactose-fermenting, non-hydrogen producing bacteria (e.g. Bifidobacteria) [20]. Diarrhea due to LI occurs mainly in infants and young children as this age group lacks the ability to compensate by colonic reabsorption. In older children, reabsorption of fermentation products (e.g. SCFA, lactate) reduces the osmotic load and significantly reduces diarrhea. Diarrhea after smaller amounts of milk should therefore not simply be attributed to LI alone, and other medical causes (e.g. non-IgE-mediated CMA) be considered [21].

## Genetics and epidemiology of lactose intolerance

The lactase gene is located on the long arm of chromosome 2 (region 2q21) [22]. Its expression is regulated by a promoter region located upstream from the gene. Maximum lactase expression in enterocytes occurs during the first months of life and declines after weaning [23, 24]. In individuals with LNP, lactase levels gradually fall to about 10–25% compared to those of young infants due to a decrease in mRNA [6, 18].

Several single nucleotide polymorphisms (SNP) have been identified in the promoter region of the lactase gene [25]. The most common polymorphism associated with lactase persistence in Caucasians is characterized by a C > T change at 13910 base pairs upstream of the lactase gene. Several other polymorphisms for lactose persistence have been identified with specific regional differences [26–31]. While the C/C_13910_ genotype is associated with lactose malabsorption, the genotypes C/T and T/T are found in individuals with lactase persistence [2]. Heterozygotes carrying the C/T allele differ in their response to an oral lactose load, compared to C/C and T/T genotypes, suggesting an intermediate phenotype [32].

Lactase persistence is common in people of Northern European, West African or Middle Eastern background. Estimated prevalence figures for primary LI due to LNP are 2–5% in Northern Europe (Scandinavia, Germany, Great Britain), 17% in Finland and Northern France, about 50% in South America and Africa, and between 90 and 100% in Southeast Asia [33]. In North American adults, the rates of LI vary by ethnicity (79% of Indigenous Americans, 75% of African-Americans, 51% of Hispanics, and 21% of Caucasians) [6, 34].

## Physiological benefits of lactose in human milk

Lactose in human milk contributes significantly to the daily energy intake of breastfed infants. Due to the required hydrolysis by lactase, there is a delayed and sustained effect on blood glucose levels. Lactose in breast milk is thought to increase the absorption of calcium [35]. As young infants do not absorb all of the lactose from breast milk, malabsorbed lactose acts as a prebiotic [36]. This is associated with increased counts of Bifidobacteria and increased concentrations of SCFA which confer a protective effect on colonic mucosal integrity and have a beneficial effect on early immune development [37].

## Definitions and classifications

It is important to distinguish between the terms lactase deficiency, lactose malabsorption and lactose intolerance which are often used interchangeably. ‘Lactase deficiency’ describes the state of reduced lactase expression, compared to term infants. ‘Lactose malabsorption’ indicates that not all ingested lactose was absorbed and that some has reached the large intestine. ‘Lactose intolerance’ is clinically defined as lactose malabsorption with associated gastrointestinal symptoms. There are four main clinical types of LI: developmental lactase deficiency, congenital lactase deficiency (alactasia), lactase non-persistence (LNP) and secondary lactose intolerance (Table 1).

**Table 1:** Clinical classification of lactose intolerance

Developmental lactase deficiency	Observed in premature infants (less than 34 weeks of gestation) due to temporary lactase deficiency which improves with time. The peak lactase expression is reached at term when an infant typically tolerates up to 60-70 g of lactose per day, corresponding with one liter of breast milk.
Congenital lactase deficiency (alactasia)	Rare and severe autosomal recessive disorder presenting in newborn infants with severe osmotic diarrhea at commencement of breast feeding. Case reports are mainly from Finland and Western Russia. Small intestinal lactase activity is completely absent. The small intestinal mucosa is otherwise normal.
Lactase non-persistence (hypolactasia)	Physiological gradual decline of lactase activity after weaning. This occurs in about 70% of the global population. Significant gastrointestinal symptoms generally do not occur before 5 years of age. The peak onset is in teenagers and young adults. Small amounts of lactose are tolerated by most affected individuals if taken in divided amounts during the day (up to 24 g per day in older children and adults).
Secondary lactose intolerance	May occur as a consequence of small bowel injury due conditions such as viral gastro-enteritis, giardiasis, celiac disease or Crohn’s disease. Rare causes of secondary lactose intolerance include epithelial dysplasia syndromes (e.g. microvillus inclusion disease, tufting enteropathy) which present with severe malabsorption and intestinal failure in early infancy. Infants with glucose-galactose malabsorption have normal lactase activity but present with osmotic diarrhea due to the inability to absorb glucose and galactose (derived from lactose).

### Developmental lactase deficiency

Lactase is the last small intestinal disaccharidase to develop during intrauterine development. In premature infants (26–34 weeks’ gestation), lactase activity reaches about 30% of that of term infants (maturational delay) [23]. When commencing breast milk or formula, premature infants may develop clinical signs of lactose malabsorption when exposed to breast milk or formula which are usually transient. A Cochrane review examining the role of enteral lactase supplementation on anthropometric measurements and gastrointestinal symptoms in premature infants found no major clinical benefits [38, 39].

### Congenital lactase deficiency (alactasia)

Alactasia is a rare and severe autosomal recessive disorder of the newborn infant [40]. The condition mainly occurs in Finland and Western Russia [41]. Infants present with watery diarrhea, flatulence and failure to thrive after commencing breast milk or formula feeding. This may lead to life-threatening dehydration or electrolyte imbalances. Lactase activity is either completely absent or very low while other duodenal disaccharidases are detected at normal levels [42]. The intestinal epithelium is histologically normal. Several mutations in the lactase gene have been described [41]. Rarely, intestinal epithelial dysplasia syndromes (e.g. microvillus inclusion disease, tufting enteropathy) or defects in the SGLT-1 transporter (e.g. congenital glucose-galactose malabsorption) may mimic congenital lactase deficiency.

### Lactase non-persistence

LNP (also called hypolactasia) is the most common cause of LI. While the declince in lactase levels starts soon after weaning, symptoms generally do not manifest before 5 years of age [43]. In an Indonesian study, the prevalence of symptomatic hypolactasia at 3 years of age was 9.1%. The prevalence rose to 28.6% at 5 years, and 73% at 12–14 years of age [44].

### Secondary lactose intolerance

Secondary LI occurs as a result of small intestinal villous damage and decreased lactase expression. In young children, the most common causes of secondary LI include viral gastroenteritis [10], giardiasis [45], non-IgE-mediated cow’s milk enteropathy [46], celiac disease [47] and Crohn’s disease [48]. Secondary LI usually resolves within 1–2 months, depending on the underlying gut disorder [49].

## Clinical presentation

The clinical presentation of LI differs significantly between infants and older children. Symptoms generally occur within 30–60 min of ingesting lactose-containing foods. Infants with lactose malabsorption are more prone to develop diarrhea, compared to older children and adults. A low fecal pH < 5.5 may cause perianal skin irritation and excoriation. While some infants with secondary LI may experience abdominal pain and distension, infantile colic is generally not caused by LI. Lactose-free formula is therefore thought to be ineffective and is not recommended for the treatment of colic [50].

In older children and adults, symptoms of LI include abdominal pain, bloating, abdominal distension, flatulence, borborygmi and low-grade diarrhea. In a case series of 98 Indonesian adolescents with LI, abdominal pain was the main complaint (64.1%), followed by abdominal distension (22.6%), nausea (15.1%), flatulence (5.7%) and diarrhea (1.9%) [44]. In adolescents and adults, irritable bowel syndrome-like symptoms are often perceived to be due to LI. However, subsequent double-blind challenges have failed to demonstrate a clear benefit of lactose restriction in these patients [51]. LI is therefore thought to be a contributing factor in irritable bowel syndrome, but visceral hyperalgesia and reactions to other fermentable carbohydrates may also be of importance [52].

## Laboratory diagnosis

The diagnosis of LI relies on the observation of gastrointestinal symptoms after ingestion of lactose-containing foods, including breast milk, cow’s milk or other mammalian milk. Several diagnostic methods are available to confirm lactose malabsorption. In children, causes of secondary LI should always be considered in the differential diagnosis.

### Reducing sugars and pH in stool

Measurement of total and reducing sugars in stool is an indirect test for lactose malabsorption [53]. Apart from lactose, other reducing sugars (e.g. glucose, galactose and fructose) are also detected by this method. The test is therefore not specific for LI. The stool pH in infants with LI is typically below 5.5 to 6.0. The liquid portion of a stool sample should be analyzed. Up to 0.25% of total/reducing sugars is considered normal. In young breastfed infants with physiological lactose malabsorption, concentrations may be higher [54]. The test is not recommended in children older than 2 years of age due to a righ rate of false-negative results.

### Lactose breath hydrogen testing

Breath hydrogen testing relies on the detection of exhaled hydrogen after a standard dose of lactose. After an overnight fasting period, baseline hydrogen should be close to 0 parts per million (ppm). Breath samples are taken every 15–30 min for 3 h (from time of the lactose bolus). A rise in exhaled hydrogen by ≥20 ppm from baseline is considered diagnostic [55]. False negative result may occur in the absence of hydrogen producing bacteria, e.g. after recent antibiotic treatment. For this reason, a positive control test with lactulose (a non-absorbable synthetic disaccharide) is required for validation of a negative result to lactose. Another method of reducing the risk of false-negative results is the co-measurement of exhaled methane [56, 57]. Both hydrogen and methane are bacterial breakdown products from lactose. Exhaled methane levels rise after the bacterial fermentation of malabsorbed lactose, even if hydrogen-producing bacteria are absent. A rise in exhaled methane by ≥10 ppm from baseline is considered evidence of lactose malabsorption [55]. The correlation of breath hydrogen testing (BHT) results with clinical symptoms is variable. While low-grade diarrhea and flatulence during the BHT are highly specific symptoms, abdominal pain on its own should not be attributed to LI [58].

### Duodenal disaccharidases

Lactase and other duodenal disaccharidases (sucrase, maltase, isomaltase) are measured in duodenal biopsies which can be obtained during gastroscopy. In cases of cow’s milk enteropathy or celiac disease with villous damage, lactase concentrations are typically reduced while sucrase levels are sufficient [47, 59]. In infants with congenital alactasia, lactase is either very low or completely absent while the histological appearance of the duodenum is normal [42].

### Genetic diagnosis of hypolactasia

Genetic testing allows the prediction of hypolactasia, even before symptoms are present. Commercial assays are based on the C > T_13910_ polymorphism which is associated with lactase persistence in Caucasians. Testing for other SNPs may also become available. The clinical usefulness of this test is controversial as it may lead to unnecessary lactose restriction before symptoms are present.

## Treatment of lactose intolerance

In infants with LI, breast-feeding should be continued. In formula-fed infants, a limited trial of lactose-free formula may be indicated, e.g. following viral gastroenteritis. In children with persistent diarrhea following acute gastroenteritis, lactose restriction has been shown to shorten the duration of gastrointestinal symptoms [11]. Reintroduction of lactose-containing formula or foods should be attempted after 2–4 weeks, as tolerated. In infants with celiac disease or other small intestinal pathology, lactose restriction may be required until the underlying condition has resolved or been adequately treated. This also applies to infants with non-IgE-mediated CMA and enteropathy. In these infants, a lactose-containing EHF is often tolerated after the gut pathology has resolved on a hypoallergenic elimination diet [46].

In individuals with LI, lactose-containing foods should be reduced but do not need to be eliminated completely. Adolescents and adults with hypolactasia tolerate up to 12-24 g of lactose daily, if taken in divided amounts. Consuming milk with a meal and in divided doses improves overall tolerance as it slows the release of lactose in the small intestine. Dietary lactose is mainly derived from fresh cow’s milk and other milk-based dairy products (e.g. yoghurt, ice cream). The content in yoghurt is lower than in milk due to breakdown of lactose by lactose-fermenting bacteria. As lactose is mainly found in the watery portion of milk, hard cheese only contains small amounts (0.1 to 0.9 g in 30 g of hard cheese), and the lactose content of butter is negligible [21]. Some medications contain lactose as a carrier, but amounts are rarely sufficient to be clinically relevant [60].

### Treatment of infants with congenital lactose deficiency (alactasia)

In infants with congenital lactase deficiency, breast milk or lactose-containing formula cause persistent watery diarrhea and growth failure. In this situation, breast feeding can generally not be sustained. Infants with congenital lactase deficiency require a change to a lactose-free formula. If recognized and treated early, infants with congenital lactase deficiency achieve normal growth and development [61]. Lactose restriction needs to be continued for life. However, older children and adults may tolerate small amounts of dietary lactose, depending on the disease severity.

### Lactase supplementation

In children and adults with LI, oral lactase supplements have been shown to ameliorate the severity of gastrointestinal symptoms after a lactose challenge [62, 63]. Ingested lactase is easily broken down by gastric acid and inactivated. Lactase treatment in infants is therefore only effective if added to expressed breast milk or formula for several hours before feeding [64].

### Inappropriate use of lactose-free or lactose-reduced formula in infants with CMA

A recent survey in Northern Ireland (2012–2014) assessed formula prescription patterns in infants with likely non-IgE-mediated CMA. The survey found that thickened anti-regurgitation formula, lactose-reduced partially hydrolyzed formula or lactose-free, cow’s milk protein-containing formulas were commonly prescribed in infants with symptoms suggestive of non-IgE-mediated CMA [7]. The survey was repeated after the implementation of active education and national feeding guidelines [7]. Following the educational interventions, the use of EHF and AAF increased by 63%, while the use of alternative treatments was reduced by 44.6%. Overall, the recognition of CMA increased from 3.4% to 9.8% of treated infants. This study demonstrates both the need for health care provider education on gastrointestinal CMA, as well as the usefulness of educational campaigns and national treatment guidelines.

### Role of extensively hydrolyzed formula with lactose in infants with CMA

**Fig. 1:**
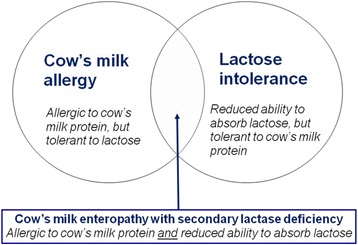
Clinical overlap between cow’s milk allergy and lactose intolerance

Extensively hydrolyzed formula (EHF), the first-line treatment for formula-fed infants with CMA, was initially designed to treat malabsorption. For this reason, early generation EHF were typically lactose-free and short peptide-based formulas with a high content of medium chain triglycerides (MCT). In recent years, lactose has been added to extensively hydrolyzed formula (EHF). Lactose in hydrolyzed formula has been shown to increase the absorption of calcium, when compared to lactose-free formula [35]. Highly purified lactose is tolerated well by cow’s milk-allergic infants [65, 66]. Lactose restriction is only warranted in infants with CMA if an enteropathy with secondary lactase deficiency is present (Fig. 1). Lactose may cautiously be reintroduced after about 1–2 months, once symptoms have resolved and small intestinal lactase activity been restored. Table 2 summarizes the various subtypes of IgE- and non-IgE-mediated CMA in relation to lactose restriction.

**Table 2:** Lactose restriction in cow’s milk allergic infants

Type of cow’s milk allergy (CMA)	Need for lactose restriction
IgE-mediated CMA / anaphylaxis	NO ^a^
Cow’s milk protein-induced enteropathy	YES ^b^
Cow’s milk protein-induced enterocolitis syndrome (FPIES)	NO
Cow’s milk protein-induced proctocolitis	NO
CMA-associated gastro-esophageal reflux disease	NO
CMA-associated constipation	NO
CMA-associated eczema	NO

^a^ *For formula-fed infants with non-anaphylactic IgE-mediated CMA, a lactose-containing extensively hydrolyzed formula is suitable* [60]. *In infants with a history of anaphylaxis to cow’s milk protein, an amino acid-based formula is recommended. No lactose-containing amino acid-based formula is currently available*

^b^ *For formula-fed infants with cow’s milk protein-induced enteropathy, a lactose-free extensively hydrolyzed formula or amino acid-based formula are considered the first line treatment, depending on clinical features and severity* [60]. *Lactose may be tolerated later in the treatment course after intestinal mucosal repair has been achieved on a cow’s milk protein-free diet*

The addition of lactose slightly increases the sweetness of EHF which is thought to improve the overall palatability. This reduces the risk of taste aversion and formula refusal, particularly by older infants [67]. In addition, recent studies have demonstrated that lactose in EHF confers prebiotic benefits in infants with CMA [36, 68]. The addition of lactose to EHF significantly increased the counts of Bifidobacteria, lactic acid bacteria and decreased Bacteroides and Clostridia, compared to lactose-free EHF [36]. The same study also demonstrated a positive effect on the fecal metabolome with increased concentrations of SCFA (mainly acetic and butyric acid). These prebiotic effects of lactose are likely to have a positive effects on early immune development [69]. The authors speculate that lactose may also play a role in the acquisition of tolerance, although no data to this effect are currently available [36]. Given the positive effects on fecal microbiome and metabolome, lactose-containing EHF may offer clinical and immunological benefits in the treatment of infants with CMA.

### Nutritional adequacy of lactose-free diets

Over the past decade there has been a sharp decline in the consumption of fresh cow’s milk and increased consumption of lactose-free milk and cereal milks in the community [70]. Parents may restrict milk products in their children due to unfounded concerns about LI or CMA. A study of Swedish children and adolescents assessed the likelihood of milk avoidance according to genetic lactase persister status [71]. While LI in adolescents did not affect vitamin D levels or anthropometric variables, it was associated with reduced milk and calcium intakes, compared to those who tolerated lactose (OR 3.2; 95% CI 1.5, 7.3) [71].

The main adverse health effects of LI occur as a result of milk avoidance and reduced calcium intakes. Avoidance of dairy products may lead to nutritional rickets in young children [72], as well as low bone mineral density and increased fracture risk later in life [73]. Calcium intake is a marker for dietary adequacy and closely correlates with the intake of other micronutrients [74]. Calcium absorption in individuals with LI is normal, which means that calcium can be administered as an oral supplement in non-dairy formats [75].

## Conclusion

Confusion between CMA and LI may lead to a delayed diagnosis of CMA, as well as inappropriate dietary interventions. Primary LI in children under 5 years is uncommon, even in regions with a high prevalence of primary hypolactasia. In young children with LI, an underlying gut condition should therefore always be considered in the diagnostic process. In these cases, lactose restriction is only required until the underlying condition has either resolved or been treated. Gastrointestinal CMA represents the main differential diagnosis to LI in infancy. Contrary to common belief, most infants with CMA can tolerate dietary lactose. Lactose-containing EHF offers potential benefits in the treatment of formula-fed infants with CMA due to prebiotic effects on fecal microbiome and metabolome. Evidence-based educational health campaigns are needed to address the knowledge gaps and misconceptions around LI and CMA in the community.

## Abbreviations

AAF: Amino acid-based formula
CMA: Cow’s milk allergy
EHF: Extensively hydrolyzed formula
LI: Lactose intolerance
LNP: Lactase non-persistence
MCT: Medium chain triglyceride
ppm: Parts per million
SCFA: Short chain fatty acid
SNP: Single nucleotide polymorphism
